# Heuristic Strategies for Persuader Selection in Contagions on Complex Networks

**DOI:** 10.1371/journal.pone.0169771

**Published:** 2017-01-10

**Authors:** Peng Wang, Li-Jie Zhang, Xin-Jian Xu, Gaoxi Xiao

**Affiliations:** 1 College of Sciences, Shanghai University, Shanghai 200444, China; 2 Key Laboratory of Embedded System and Service Computing (Tongji University), Ministry of Education, Shanghai 201804, China; 3 School of Electrical and Electronic Engineering, Nanyang Technological University, Singapore, Singapore; Universidad Rey Juan Carlos, SPAIN

## Abstract

Individual decision to accept a new idea or product is often driven by both self-adoption and others’ persuasion, which has been simulated using a double threshold model [Huang et al., Scientific Reports 6, 23766 (2016)]. We extend the study to consider the case with limited persuasion. That is, a set of individuals is chosen from the population to be equipped with persuasion capabilities, who may succeed in persuading their friends to take the new entity when certain conditions are satisfied. Network node centrality is adopted to characterize each node’s influence, based on which three heuristic strategies are applied to pick out persuaders. We compare these strategies for persuader selection on both homogeneous and heterogeneous networks. Two regimes of the underline networks are identified in which the system exhibits distinct behaviors: when networks are sufficiently sparse, selecting persuader nodes in descending order of node centrality achieves the best performance; when networks are sufficiently dense, however, selecting nodes with medium centralities to serve as the persuaders performs the best. Under respective optimal strategies for different types of networks, we further probe which centrality measure is most suitable for persuader selection. It turns out that for the first regime, degree centrality offers the best measure for picking out persuaders from homogeneous networks; while in heterogeneous networks, betweenness centrality takes its place. In the second regime, there is no significant difference caused by centrality measures in persuader selection for homogeneous network; while for heterogeneous networks, closeness centrality offers the best measure.

## Introduction

In many complex systems, small initial shocks can cascade to affect or disrupt the systems under certain circumstances. Examples include the diffusion of cultural fads [1], the outbreak of political unrest [2], and the spread of rumors [3], etc. These phenomena can be studied by contagion models [4, 5], in which inactive (or susceptible) individuals are activated (or infected) by contacts with active neighbors. Of particular importance is the threshold model, which originated from the seminal work of Schelling [6] on residential segregation, and subsequently was developed by Granovetter [7] in the study of social influences. The name of threshold stems from the step behavior; that is, an individual adopts a new opinion only if a critical fraction (the Watts model [4]) or number (the Centola-Macy model [5]) of her friends have already been activated. This required fraction/number of adopters in the neighborhood is defined as the threshold. Hereafter, we call it adoption threshold.

Although the propagation rule is simple, the threshold model can exhibit complex behavior when individual heterogeneity and interaction structure are considered. Watts [4] studied the model with one random initiator on complex networks to examine the effects of two factors on the cascade dynamics: it was found that heterogeneous nodal degrees enhance systemic stability compared to that of homogeneous networks. Threshold heterogeneity, however, has an opposite effect. Gleeson and Cahalane [8] extended Watts’ model to a finite number of initiators. The varying seed size has an effect on the cascade transition as a function of the average nodal degree *z*, even making the transition to be discontinuous for relatively small values of *z*. Following this line, a series of studies have been carried out by considering other network properties, such as degree correlation [9, 10], weight [11], small world [12], modularity [13], clustering [14, 15], temporality [16, 17], multi-layers [18–20], etc.

Research has also been conducted on contagion mechanisms. Dodds and Watts [21, 22] proposed a generalized contagion model incorporating individual memory, variable magnitude of exposure, and susceptibility heterogeneity. Another study [23] decomposed the motivation for a node to adopt a new behavior as a combination of personal preference, the average of the states of each node’s neighbors and the system average. It is worth mentioning that Melnik et al. [24] considered the threshold model with multi-stages and found that global cascades can be driven not only by high-stage influencers but also by low-stage ones. Ruan et al. [25] considered individual conservativeness and studied Watts’ model with mechanisms of spontaneous adoption and complete reluctance to adoption. More recently, Huang et al. [26] considered asymmetric interactions of social networks, where the change of individual opinion depends on both catching and giving dynamics. In analogy to the catching dynamics described by the adoption threshold, the persuasion threshold was introduced to describe the giving dynamics; in other words, an activated individual can convince her inactivate friends if the active fraction among her friends is larger than a critical fraction.

In the real world, however, persuasion is more difficult than adoption. Not everyone can be a persuader, and not each persuader can succeed. An important problem is to optimize the selection of persuaders, i.e., to choose persuaders for maximizing the cascade size. To address this issue we compare three different strategies for selecting a set of persuaders with a predefined size on complex networks. Intuitively, the selection of persuaders is related to the influences of individuals in social networks [27], which can typically be measured by network nodes’ centralities, including degree centrality (DC) [28], eigenvector centrality (EC) [29], betweenness centrality (BC) [30], closeness centrality (CC) [28], and so on. With these quantities, we pick out persuaders with maximum, medium, and minimum centralities, respectively. As will be seen below, the best strategy depends on the global connectivity of the underline network. Specifically, as network connectivity varies, there exist different optimal selection strategies where persuaders should be selected with different centrality measures within different ranges. Notably, in dense networks, it leads to better performance by selecting nodes with medium rather than maximum centrality values. Moreover, in sparse homogeneous networks, selecting nodes with maximum degree centrality as persuaders performs the best, while for sparse heterogeneous networks, betweenness centrality is the measure that should be adopted; in dense homogeneous networks, all centrality measures work equally good as long as nodes with medium centralities are selected as persuaders; while in dense heterogeneous networks, nodes with medium closeness centrality should be selected.

## Methods

### Construction of interaction networks

The homogeneous networks used are Erdős-Rényi (ER) graphs [31] which can be constructed as follows. Starting with *N* isolated nodes, we connect each pair of nodes with a link with the identical probability *p*. A ER network is generated randomly from the collection of all graphs which have *N* nodes and *pN*(*N* − 1)/2 edges. The nodal degree of the ER network takes the form of the Possion distribution *P*(*k*) = *e*^−*z*^ *z*^*k*^/*k*!. The heterogeneous networks used are scale-free (SF) networks. Following the idea proposed by Newman et al. [32], the random SF network can be constructed by the following steps: i) A priori random integers sequence, each of which represents the degree of a node, is drawn from a power-law distribution *P*(*k*) = *ck*^−*r*^, where *c* and *r* are respectively the normalized factor and the power exponent. Notice that in order to generate uncorrelated SF networks, the restriction on the maximum degree $k_{c}\left(N\right)∼\sqrt{N}$ [33] is imposed. ii) Node *i* with degree *k*_*i*_ is picked out randomly from the sequence and connected to others until its degree quota *k*_*i*_ is realized. Duplicate connections are avoided. This process is repeated throughout all the elements of the sequence, and finally a network is generated randomly from the set of all graphs with the same degree sequence. All the networks we use are undirected and unweighted.

### Formulation of the (*ϕ*, *ϕ*′)-threshold model

According to Ref. [26], the (*ϕ*, *ϕ*′)-threshold model has two thresholds: the adoption threshold *ϕ* and the persuasion threshold *ϕ*′. Initially, a fraction *ρ*_0_ of nodes are chosen randomly from the network to be active, and the others are inactive. At each time step, an inactive node *i* will be activated if either of the following two conditions is satisfied: i) the active fraction of the neighbors of node *i* is larger than its adoption threshold *ϕ*_*i*_, which is defined as the adoption dynamics; or ii) the adoption dynamics does not occur, but there is at least one active neighbor *j* of the node *i* being a persuader *and* the active fraction in the neighborhood of node *j* is larger than the persuasion threshold $\phi_{j}^{\prime }$. We call it persuasion dynamics. Once a node is activated, it remains active. The system evolves according to the above rules until no further activation occurs.

### Heuristic strategies for persuader selection

Considering limited persuasion, a fraction 10% of all nodes are chosen to have persuasion capabilities who can persuade their inactive neighbors if the condition ii) is satisfied. Note that the percentage of persuaders may change while all the main conclusions would still hold. The selecting process is related to the node’s influence. As aforesaid, we use the concept of centrality to represent the node’s influence in the network. For comparison, four centrality measures (DC, EC, BC, and CC) are adopted based on which three heuristic strategies are applied to pick out a given number of persuaders: i) selecting nodes in descending order of their centrality (Cmax), ii) selecting nodes with medium centrality (Cmed), and iii) selecting nodes in increasing order of their centrality (Cmin). For the Cmed strategy, we firstly choose nodes with mean centrality. If the chosen number doesn’t reach the proportion, we then select nodes form both sides of the mean centrality as a complement. In spite of the simplicity of such heuristics, diverse selection strategies do make the dynamics much richer.

### Tree-like approximation of the threshold model

For analytical calculation, we apply the method of Ref. [26]. Given an uncorrelated network of *N* nodes following the degree distribution *P*(*k*), a fraction *ρ*_0_ of nodes are chosen randomly to be active. According to the model definition, we obtain the stable fraction of inactive nodes:

$$\eta =\left(1-\rho_{0}\right)\sum_{k_{i}=0}^{k_{max}}P\left(k_{i}\right)\sum_{s=0}^{k_{i}}C_{k_{i}}^{s}{\left(1-\alpha -\beta \right)}^{s}\beta^{k_{i}-s}F\left(\frac{k_{i}-s}{k_{i}}\right),$$ (1)

where *F*(*x*) denotes the probability that the adoption threshold *ϕ* of a node is no less than *x*. *α* represents the probability that a random neighbor *j* of the inactive node *i* is active and is chosen as a persuader. *β* represents the probability that a random neighbor *j* of the inactive node *i* is active and the active fraction in the neighborhood of *j* is less than the persuasion threshold $\phi_{j}^{\prime }$. Following the ideas of Refs. [8, 34], we obtain the self-consistent equations for the two probabilities:

$$\begin{array}{ccc} \alpha & = & \rho_{0}\sum_{k_{j}=k_{v}}^{k_{u}}Q(k_{j})\sum_{s=0}^{k_{j}}C_{k_{j}}^{s}{\left(1-\delta \right)}^{s}\delta^{k_{j}-s}\;[1-G\left(\frac{k_{j}-s}{k_{j}+1}\right)]\\ & & +(1-\rho_{0})\sum_{k_{j}=k_{v}}^{k_{u}}Q(k_{j})\sum_{s=0}^{k_{j}}C_{k_{j}}^{s}{\left(\alpha +\beta \right)}^{k_{j}-s}\;[1-F\left(\frac{k_{j}-s}{k_{j}+1}\right)]\\ & & \times \sum_{m=0}^{s}C_{s}^{m}{\left(1-\alpha -\beta -\gamma \right)}^{m}\gamma^{s-m}\;\left[1-G\left(\frac{k_{j}-m}{k_{j}+1}\right)\right]\\ & & +(1-\rho_{0})\sum_{k_{j}=k_{v}+1}^{k_{u}}Q(k_{j})\sum_{s=0}^{k_{j}-1}C_{k_{j}}^{s}\left[{\left(\alpha +\beta \right)}^{k_{j}-s}-\beta^{k_{j}-s}\right]\;F\;\left(\frac{k_{j}-s}{k_{j}+1}\right)\\ & & \times \sum_{m=0}^{s}C_{s}^{m}{\left(1-\alpha -\beta -\gamma \right)}^{m}\gamma^{s-m}\;\left[1-G\left(\frac{k_{j}-m}{k_{j}+1}\right)\right], \end{array}$$ (2)

$$\begin{array}{c} \beta =1-\alpha -\left(1-\rho_{0}\right)\sum_{k_{j}=0}^{k_{max}}Q\left(k_{j}\right)\sum_{s=0}^{k_{j}}C_{k_{j}}^{s}{\left(1-\alpha -\beta \right)}^{s}\beta^{k_{j}1-s}F\;\left(\frac{k_{j}-s}{k_{j}+1}\right), \end{array}$$ (3)

where *G*(*x*) denotes the probability that the persuasion threshold *ϕ*′ of a node is no less than *x*. *Q*(*k*) ≡ (*k* + 1)*P*(*k* + 1)/*z* is the excess degree distribution. *k*_*u*_ and *k*_*v*_ correspond to upper and lower bounds of degrees of the nodes that have been selected as persuaders, respectively. In Ref. [26], all the nodes are potential persuaders, and the upper and lower bounds are maximum and minimum degrees, respectively. *γ* refers to the critical case separating *α* and *β*. *δ* describes the probability that a random neighbor *j* of the active node *i* is active, written as

$$\begin{array}{ccc} \gamma & = & \left(1-\rho_{0}\right)\overset{k_{max}}{\underset{k_{j}=0}{\sum }}Q\left(k_{j}\right)\overset{k_{j}}{\underset{s=0}{\sum }}C_{k_{j}}^{s}{\left(1-\alpha -\beta \right)}^{s}\beta^{k_{j}-s}\\ & & \times \left[F\left(\frac{k_{j}-s}{k_{j}}\right)-F\left(\frac{k_{j}+1-s}{k_{j}+1}\right)\right], \end{array}$$ (4)

$$\begin{array}{c} \delta =1-\left(1-\rho_{0}\right)\sum_{k_{j}=0}^{k_{max}}Q\left(k_{j}\right)\sum_{s=0}^{k_{j}}C_{k_{j}}^{s}{\left(1-\alpha -\beta \right)}^{s}\beta^{k_{j}-s}F\;\left(\frac{k_{j}+1-s}{k_{j}+1}\right). \end{array}$$ (5)

One can solve the above equations using a simple iterative scheme, and finally get the stable size of the giant component of inactive nodes:

$$\eta_{c}=\left(1-\rho_{0}\right)\sum_{k_{i}=1}^{k_{max}}P\left(k_{i}\right)\sum_{s=1}^{k_{i}}C_{k_{i}}^{s}\beta^{k_{i}-s}F\;\left(\frac{s}{k_{i}}\right)\;\sum_{m=1}^{s}C_{s}^{m}{\left(1-\alpha -\beta -\theta \right)}^{m}\theta^{s-m},$$ (6)

where *θ* is the probability that a random neighbor *j* of the inactive node *i* is inactive but not belonging to the giant component of the inactive nodes, given by

$$\theta =\left(1-\rho_{0}\right)\sum_{k_{j}=0}^{k_{max}}Q\left(k_{j}\right)\sum_{s=0}^{k_{j}}C_{k_{j}}^{s}\theta^{s}\beta^{k_{j}-s}F\;\left(\frac{k_{j}-s}{k_{j}+1}\right).$$ (7)

## Results

The persuasion rule characterizes the situation that a persuader convinces her friends to accept the new entity. Therefore it gives rise to global cascades. According to the model definition, the higher the value of *ϕ*′, the lower the persuasion possibility is. In the extreme case *ϕ*′ = 1, the double threshold model reduces to Watts’ model [4]. In this scenario, the cascade condition in random networks for one seed is ∑_*k*_ *k*(*k* − 1)*ϱ*(*k*)*P*(*k*) = *z*, where *ϱ*(*k*) represents the distribution of vulnerable nodes. While the network is sparse, the criterion of the cascade is *ϕ* < 1/*z*. But if the number of initiators is sufficiently large, large cascades will occur irrespective of the value of *ϕ* [8, 35, 36].

### Impact of selection strategies on the cascade dynamics

**Fig 1:**
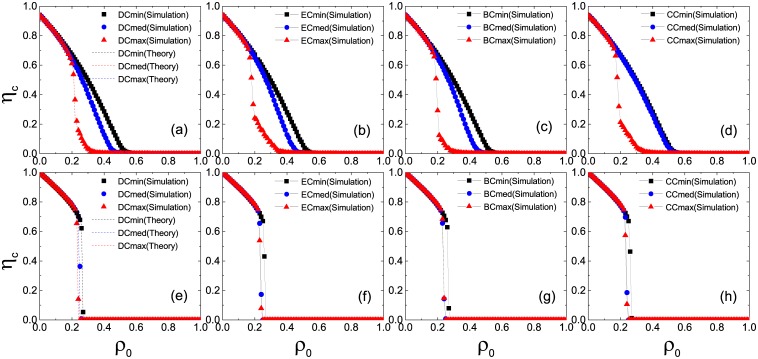
Normalized size of the giant component of inactive nodes *η*_*c*_ in the stable state as a function of the seed fraction *ρ*_0_ in homogeneous networks. Symbols represent simulation results on ER networks of *N* = 10^4^ nodes and average degree *z* = 3 (upper panel) and 10 (lower panel), respectively. All the results are averaged over 10 realizations of the model, each of which is performed on 10 network configurations. Dashed lines are theoretical predictions by Eq (6). DC, EC, BC, and CC correspond to degree centrality, eigenvector centrality, betweenness centrality, and closeness centrality, respectively.

**Fig 2:**
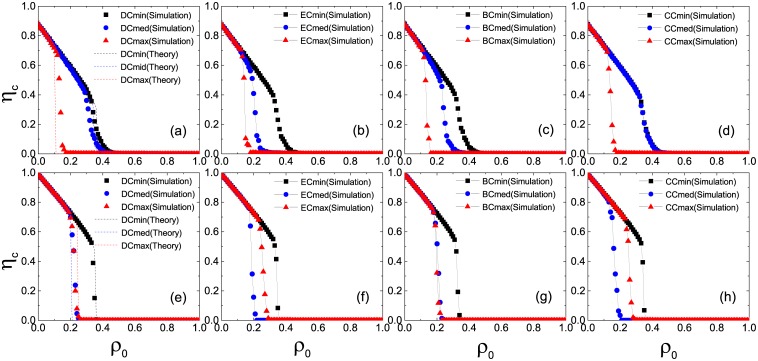
Normalized size of the giant component of inactive nodes *η*_*c*_ in the stable states as a function of the seed fraction *ρ*_0_ in heterogeneous networks. Symbols represent simulation results on SF networks of *N* = 10^4^ nodes and average degree *z* = 3 (upper panel) and 10 (lower panel), respectively. All the results are averaged over 10 realizations of the model, each of which is performed on 10 network configurations. Dashed lines are theoretical predictions by Eq (6).

In Fig 1 we plot the normalized size of the final giant component of inactive nodes *η*_*c*_ as a function of the seed fraction *ρ*_0_ in the ER network. Without loss of generality, we let *ϕ* = *ϕ*′ = 0.5 (Note that all the main conclusions hold for other cases we have tested as well.). From left to right, the selection strategies are based on the DC, EC, BC, and CC, respectively. All the plots separate two phases, defining the transition point *ρ*_*c*_. Global cascades are observed when *ρ*_0_ > *ρ*_*c*_. In case of *z* = 3 (upper panel), one can find that the Cmax strategy (closed triangles) is optimal to make the system most vulnerable, since it needs the smallest size of initiators to trigger a large cascade. In case of *z* = 10 (lower panel), the Cmed strategy, as well as the Cmax strategy, have a marginal advantage over the Cmin strategy. Meanwhile, the system exhibits discontinuous transitions with a sharp drop from a finite size to zero at *ρ*_*c*_. Fig 2 shows plots of *η*_*c*_ as a function of *ρ*_0_ in the SF network. Both adoption and persuasion thresholds are the same as those in Fig 1. In case of *z* = 3 (upper panel), one notices similar behaviors, i.e., the optimal method to increase the likelihood of global cascades is the Cmax strategy (closed triangles). In case of *z* = 10 (lower panel), however, the Cmed strategy (closed circles) becomes superior in causing global cascades except for the BC case where the Cmax strategy performs equally well (Fig 2(g)).

**Fig 3:**
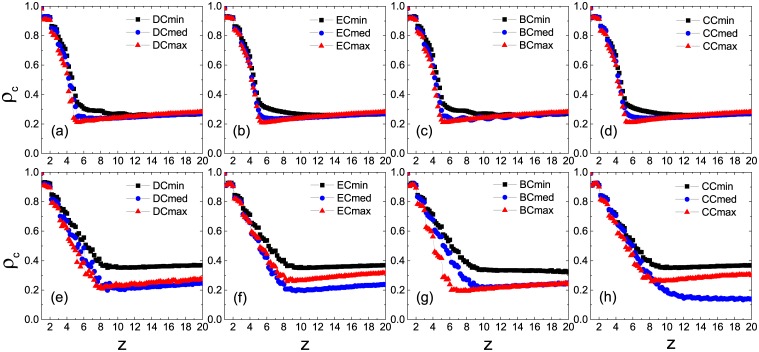
Minimum seed fractions *ρ*_*c*_ for causing global cascades as a function of the average node degree *z*. All the results are averaged over 10 realizations of the model, each of which is performed on 10 ER (upper panel) and SF (lower panel) network configurations, respectively.

**Fig 4:**
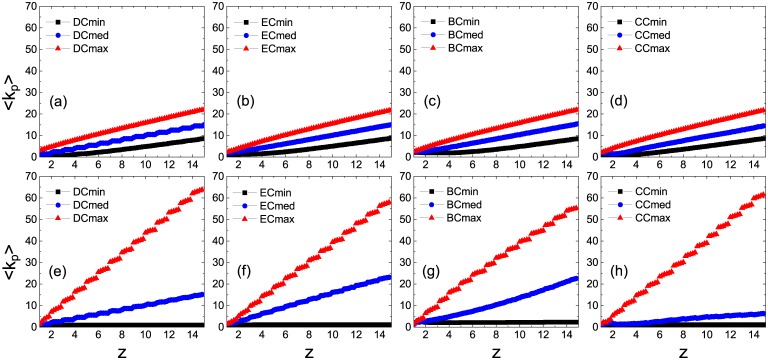
Average degree of selected persuaders 〈*k*_*p*_〉 as a function of the average degree of total nodes *z*. All the results are averaged over 10 realizations of the model, each of which is performed on 10 ER (upper panel) and SF (lower panel) network configurations, respectively.

To draw a general view of the above results, we plot in Fig 3 the minimum fraction *ρ*_*c*_ of initial seeds for causing global cascades as a function of the average node degree *z*. In the ER network (upper panel), the plots of the Cmax strategy (closed triangles) are lowest when network connectivity is sufficiently sparse, hence the optimal solution to promote the cascade dynamics. While the connectivity is sufficiently dense, the Cmed strategy takes its place with a little advantage. This conclusion holds in the SF network as well (lower panel) except for the BC measure [Fig 3(g)] where the Cmax strategy performs equally well as the Cmed strategy. To achieve further insights, we calculate the average node degree of selected persuaders 〈*k*_*p*_〉 as a function of *z* in Fig 4. In the ER network (upper panel), 〈*k*_*p*_〉 increases linearly with *z* under three selection strategies and the increasing rates are relatively low. In the low-connectivity regime, the cascade propagation is limited by the global connectivity of the network. As the average degree of selected persuaders under three selection strategies is several times as much as the network connectivity, they give rise to global cascades more easily. Among these operations, the value of 〈*k*_*p*_〉 is largest under the Cmax strategy, implying optimal persuasion. In the high-connectivity regime, the cascade propagation is limited by local stability of individual nodes. As a persuader is surrounded by many inactive neighbors, there is a lower chance for her to satisfy the persuasion threshold. Therefore the difference in the three strategies is small. In the SF network (lower panel), however, the difference in 〈*k*_*p*_〉 is obvious. The value of 〈*k*_*p*_〉 under the Cmax strategy is larger than that of the ER network. While under the Cmin strategy, 〈*k*_*p*_〉 is almost independent of *z*. The plot of the Cmed strategy lies between the cases of Cmax and Cmin strategies with the same order of the increasing rate as that on the ER network, hence having the largest inducing effect on the highly connected network.

### Impact of centrality measures on the optimal strategies

We have noticed that some generic features of global cascades can be explained in terms of network connectivity. For both the ER and SF networks, the Cmax strategy is optimal to promote global cascades in networks with low connectivity, while the Cmed strategy becomes superior in dense networks. Next, we shall probe which centrality measure is most suitable for choosing the set of persuaders while adopting the optimal selection strategies in the two different regimes, respectively.

**Fig 5:**
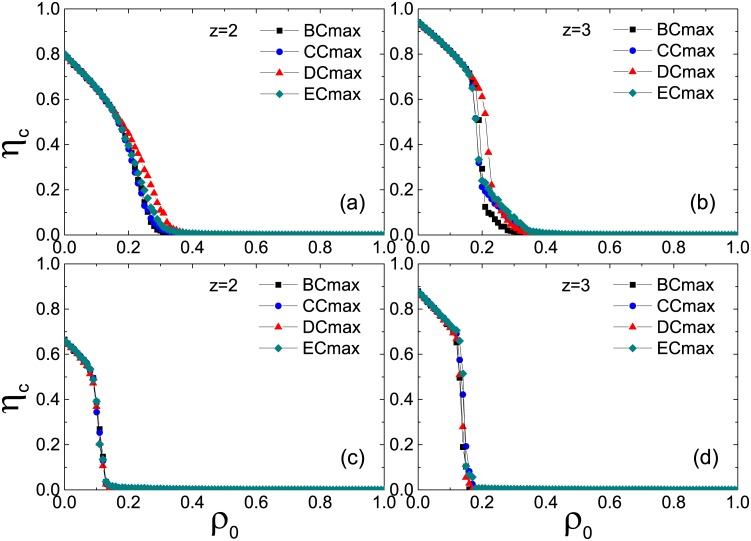
Normalized size of the giant component of inactive nodes *η*_*c*_ in the stable states as a function of the seed fraction *ρ*_0_ in the network with *z* = 2 and 3, respectively. The Cmax strategy is applied to pick out persuaders. All the results are averaged over 10 realizations of the model, each of which is performed on 10 ER (upper panel) and SF (lower panel) network configurations, respectively.

**Fig 6:**
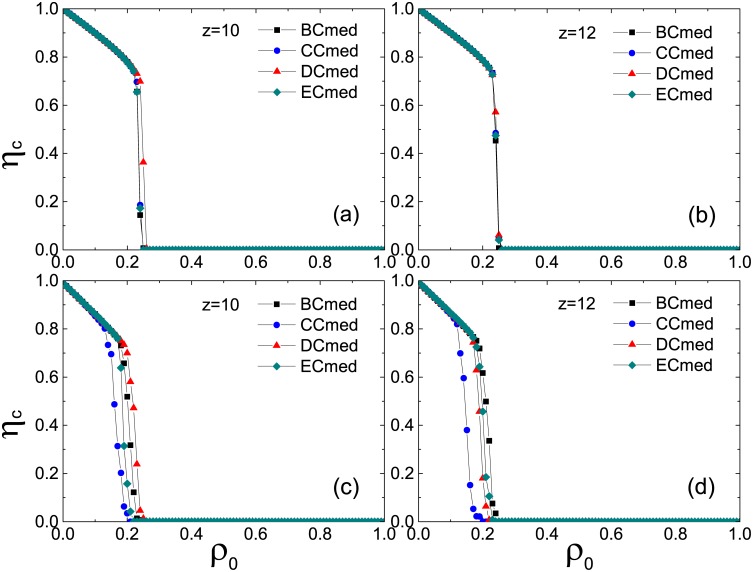
Normalized size of the giant component of inactive nodes *η*_*c*_ in the stable states as a function of the seed fraction *ρ*_0_ in the network with *z* = 10 and 12, respectively. The Cmed strategy is applied to pick out persuaders. All the results are averaged over 10 realizations of the model, each of which is performed on 10 ER (upper panel) and SF (lower panel) network configurations, respectively.

Fig 5 shows the transition behavior of *η*_*c*_ as a function of *ρ*_0_ under the Cmax strategy in cases of *z* = 2 and 3, respectively. Still let *ϕ* = *ϕ*′ = 0.5. For the ER network (upper panel), the system exhibits a continuous transition under the Cmax strategy for all the centrality measures with a little effect on the transition point; whereas for the SF network (lower panel), the unique transition point reflects the same effect of all the centrality measures on persuader selection. Fig 6 shows an opposite behavior under the Cmed strategy in cases of *z* = 10 and 12, respectively. For both networks, the system exhibits a discontinuous transition. Moreover, in contrast to the ER networks with the coincidence of all the plots (upper panel), the SF networks demonstrate diverse influences of centrality measures with CC performing best (lower panel).

**Fig 7:**
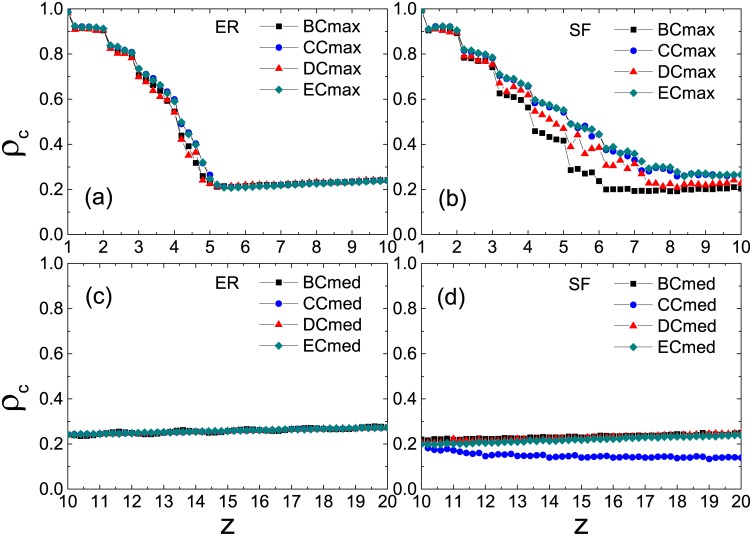
Minimum seed fractions *ρ*_*c*_ for global cascades as a function of the average degree *z* under two optimal strategies. The Cmax and Cmed strategies are used in low-connectivity (upper panel) and high-connectivity (lower panel) regimes, respectively. All the results are averaged over 10 realizations of the model, each of which is performed on 10 network configurations, respectively.

**Fig 8:**
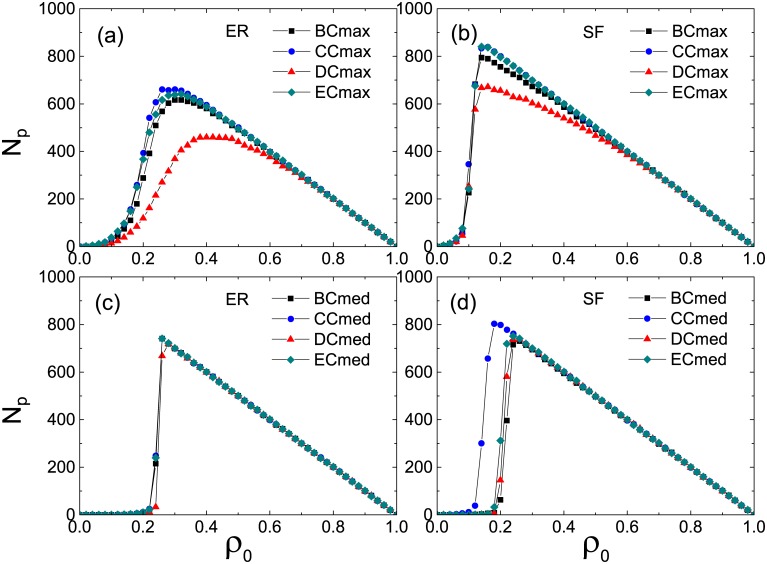
Actual number of persuaders *N*_*p*_ in the stable states as a function of the seed fraction *ρ*_0_. The Cmax and Cmed strategies are applied to the cases of *z* = 2 (upper panel) and *z* = 12 (lower panel), respectively. All the results are averaged over 10 realizations of the model, each of which is performed on 10 network configurations, respectively.

To get a clear understanding on this point, we plot *ρ*_*c*_ as a function of *z* for global cascades in Fig 7. Under the Cmax strategy in the low-connectivity regime, the value of *ρ*_*c*_ is lowest corresponding to the DC in the ER network [Fig 7(a)], indicating the best solution for persuader selection; in the SF network, however, the BC results in the lowest *ρ*_*c*_ [Fig 7(b)] in a wide range, hence the most appropriate solution. Under the Cmed strategy in the high-connectivity regime, in contrast to the case on the ER network where effects of the four centralities on the transition point are nearly the same [Fig 7(c)], the SF network turns out to be more vulnerable when CC is utilized [Fig 7(d)] to pick out persuaders. Since a potential persuader can succeed in persuading others only when the persuasion threshold is achieved, we term such persuaders as *actual persuaders*. In Fig 8, we illustrate the number of actual persuaders *N*_*p*_ as a function of *ρ*_0_ in the stable states. We see that, for all the different connectivity levels, CC always leads to a larger number of actual persuaders in the SF networks than in the ER network. Similar conclusion holds for the other three centrality measures in most cases as well.

## Discussion

Interpersonal influences of social networks are usually asymmetric, that is, the diffusion of an entity among individuals depends on both the probability of giving it and the probability of catching it. We classify the acceptance of an entity as due to self-adoption and others’ persuasion, which can be simulated by the (*ϕ*, *ϕ*′)-threshold model. Although the adoption mechanism has stimulated a rapid acceleration of research work, little attention has been paid to the persuasion mechanism. The focus of this work is to identify optimal strategies for persuader selection and study their persuasion effects and dynamics on the networks. Since the optimization selection of nodes for information maximization in a general complex network is NP-hard [37], heuristic algorithms become the most common approaches, e.g., to rank all nodes according to their degrees or centralities and choose the highest-value ones.

We utilized four centralities (DC, EC, BC, and CC) to rank each node’s influence and applied three heuristic strategies (Cmax, Cmed, and Cmin) to select 10% of network nodes as potential persuaders. We first examined the impacts of three selection strategies on the cascade dynamics. When network connectivity is sufficiently sparse, the Cmax strategy is optimal for persuader selection to promote global cascades; when it is sufficiently dense, the Cmed strategy performs best. Under optimal strategies, we studied further which centrality measure is most appropriate for persuader selection. In the low-connectivity regime, we found that the DC is most suitable for the homogeneous networks under the Cmax strategy; whereas the heterogeneous networks favors the BC. In the high-connectivity regime, all the centrality measures have nearly the same effect on the Cmed strategy for the homogeneous networks, whereas for heterogeneous networks, the CC results in largest cascade size compared with other centrality measures. We also simulated the case of 5% and obtained qualitatively same results.

Although the underline networks and heuristic algorithms are elementary, we do obtain striking results of optimal solutions. In future study, it is interesting to consider more topological and dynamical features. On the one hand, one can design efficient algorithms for identifying influential nodes in various networks, e.g., temporal networks and multi-layer networks. On the other hand, one may consider more dynamical processes, e.g., transportation and routing.

## References

[1] Bikhchandani S, Hirshleifer D, Welch I. A theory of fads, fashion, custom, and cultural change as informational cascades. J Polit Econ. 1992; 100: 992–1026. 10.1086/261849

[2] Lohmann S. The dynamics of informational cascades. World Polit. 1994; 47: 42–101. 10.2307/2950679

[3] Moreno Y, Nekovee M, Pacheco AF. Dynamics of rumor spreading in complex networks. Phys Rev E. 2004; 69: 066130 10.1103/PhysRevE.69.066130 15244690

[4] Watts DJ. A simple model of global cascades on random networks. Proc Natl Acad Sci USA. 2002; 99: 5766–5771. 10.1073/pnas.082090499 16578874 PMC122850

[5] Centola D, Macy M. Complex contagions and the weakness of long ties. Am J Sociol. 2007; 113: 702–734. 10.1086/521848

[6] Schelling TC. Hockey helmets, concealed weapons, and daylight saving. J Conflict Resolut. 1973; 17: 381–428. 10.1177/002200277301700302

[7] Granovetter M. Threshold models of collective behavior. Am J Sociol. 1978; 83: 1420–1443. 10.1086/226707

[8] Gleeson JP, Cahalane DJ. Seed size strongly affects cascades on random networks. Phys Rev E. 2007; 75: 056103 10.1103/PhysRevE.75.056103 17677129

[9] Gleeson JP. Cascades on correlated and modular random networks. Phys Rev E. 2008; 77: 046117 10.1103/PhysRevE.77.046117 18517700

[10] Dodds PS, Payne JL. Analysis of a threshold model of social contagion on degree-correlated networks. Phys Rev E. 2009; 79: 066115 10.1103/PhysRevE.79.066115 19658572

[11] Hurd TR, Gleeson JP. On Watts cascade model with random link weights. J Complex Networks. 2013; 1: 25–43. 10.1093/comnet/cnt003

[12] Centola D, Eguíluz VM, Macy MW. Cascade dynamics of complex propagation. Physica A. 2007; 374: 449–456. 10.1016/j.physa.2006.06.018

[13] Galstyan A, Cohen P. Cascading dynamics in modular networks. Phys Rev E. 2007; 75: 036109 10.1103/PhysRevE.75.036109 17500761

[14] Ikeda Y, Hasegawa T, Nemoto K. Cascade dynamics on clustered network. J Phys: Conference Series 2010; 221: 012005.

[15] Hackett A, Melnik S, Gleeson JP. Cascades on a class of clustered random networks. Phys Rev. E. 2011; 83: 056107 10.1103/PhysRevE.83.056107 21728605

[16] Karimi K, Holme P. Threshold model of cascades in empirical temporal networks. Physica A. 2013; 392: 3476–3483. 10.1016/j.physa.2013.03.050

[17] Backlund VP, Saramäki J, Pan RK. Effects of temporal correlations on cascades. Phys Rev E. 2014; 89: 062815 10.1103/PhysRevE.89.062815 25019841

[18] Brummitt CD, Lee KM, Goh KI. Multiplexity-facilitated cascades in networks. Phys Rev E. 2012; 85: 045102(R) 10.1103/PhysRevE.85.045102 22680529

[19] Yağan O, Gligor V. Analysis of complex contagions in random multiplex networks. Phys Rev E. 2012; 86: 036103 10.1103/PhysRevE.86.036103 23030976

[20] Lee KM, Brummitt CD, Goh KI. Threshold cascades with response heterogeneity in multiplex networks. Phys Rev E. 2014; 90: 062816 10.1103/PhysRevE.90.062816 25615156

[21] Dodds PS, Watts DJ. Universal Behavior in a Generalized Model of Contagion. Phys Rev Lett. 2004; 92: 218701 10.1103/PhysRevLett.92.218701 15245323

[22] Dodds PS, Watts DJ. A generalized model of social and biological contagion. J Theor Biol. 2005; 232: 587–604. 10.1016/j.jtbi.2004.09.006 15588638

[23] McCullen N, Rucklidge A, Bale C, Foxon T, Gale W. Multiparameter models of innovation diffusion on complex networks. SIAM J Appl Dyn Syst. 2013; 12: 515–532. 10.1137/120885371

[24] Melnik S, Ward JA, Gleeson JP, Porter MA. Multi-stage complex contagions. Chaos. 2013; 23: 013124 10.1063/1.4790836 23556961

[25] Ruan Z, Iñiguez G, Karsai M, Kertész J. Kinetics of Social Contagion. Phys Rev Lett. 2015; 115: 218702 10.1103/PhysRevLett.115.218702 26636878

[26] Huang WM, Zhang LJ, Xu XJ, Fu XC. Contagion on complex networks with persuasion. Sci Rep. 2016; 6: 23766 10.1038/srep23766 27029498 PMC4815014

[27] Lü L, Chen D, Ren XL, Zhang QM, Zhang YC, Zhou T. Vital nodes identification in complex networks. Phys Rep. 2016 650: 1–63. 10.1016/j.physrep.2016.06.007

[28] Freeman LC. Centrality in social networks conceptual clarification. Soc Net. 1978–1979; 1: 215–239. 10.1016/0378-8733(78)90021-7

[29] Bonacich PF. Power and centrality: A family of measures. Am J Soc. 1987; 92: 1170–1182. 10.1086/228631

[30] Freeman LC. A set of measures of centrality based upon betweenness. Sociometry 1977; 40: 35–41. 10.2307/3033543

[31] Erdős P, Rényi A. On random graphs. Publ Math Debrecen. 1959; 6: 290–297.

[32] Newman MEJ, Strogatz SH, Watts DJ. Random graphs with arbitrary degree distribution and their application. Phys Rev E. 2001; 64: 026118 10.1103/PhysRevE.64.026118 11497662

[33] Catanzaro M, Boguñá M, Pastor-Satorras R. Generation of uncorrelated random scale-free networks. Phys Rev E. 2005; 71: 027103 10.1103/PhysRevE.71.027103 15783457

[34] Zhao JH, Zhou HJ, Liu YY. Inducing effect on the percolation transition in complex networks. Nat Commun. 2013; 4: 2412 10.1038/ncomms3412 24013476

[35] Singh P, Sreenivasan S, Szymanksi BK, Korniss G. Threshold-limited spreading in social networks with multiple initiators. Sci Rep. 2013; 3: 2330 10.1038/srep02330 23900230 PMC3728590

[36] Karampourniotis PD, Sreenivasan S, Szymanski BK, Korniss G. The Impact of heterogeneous thresholds on social contagion with multiple initiators. PLoS ONE. 2015; 10: e0143020 10.1371/journal.pone.0143020 26571486 PMC4646465

[37] Kempe D, Kleinberg J, Tardos É. Maximizing the spread of influence through a social network. Proc of the 9th ACM Conf SIGKDD. 2003; 137–146.

